# Superior Vena Cava Duplication: The Red Herring of Central Line Placement

**DOI:** 10.1155/2019/6401236

**Published:** 2019-11-20

**Authors:** Karin Gunther, Carmen Lam, David Siegel

**Affiliations:** Surgery Department, Ascension Macomb-Oakland Hospital, 12000 E. Twelve Mile Rd, Warren, MI 48093, USA

## Abstract

5 million central venous access lines are placed every year in the United States, and it is a common surgical bedside procedure. We present a case of a central venous catheter placement with port for chemotherapy use, during which a duplication of a superior vena cava was discovered on CTA chest after fluoroscopy could not confirm placement of the guidewire. Due to its potential clinical implications, superior vena cava duplication must be recognized when it occurs.

## 1. Introduction

Superior vena cava (SVC) duplication is a rare anomaly that occurs in 0.3% of the general population. Due to variations in the development of the embryonic thoracic venous system, a left-sided SVC persists with the physiologic right-sided SVC. Although most patients are asymptomatic unless other cardiac anomalies exist, SVC duplication can cause confusion and anxiety during a left-sided endovascular procedure when the guidewire or catheter fails to cross midline on imaging.

In this case report, we present an incidentally discovered superior vena cava duplication during a mediport placement and discuss the appropriate workup and treatment.

## 2. Case Report

**Figure 1:**
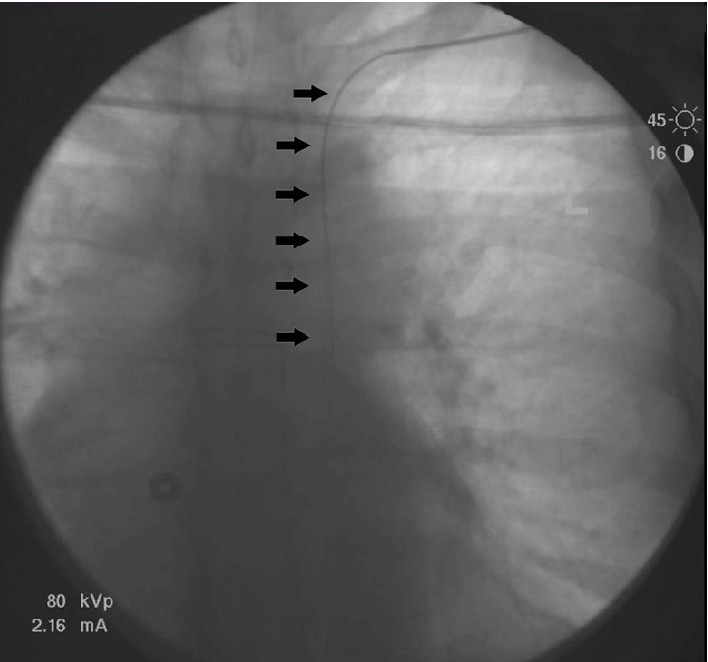
Left subclavian vein guidewire (black arrows) seen on fluoroscopy does not follow the typical anatomical course (traversing across midline) and travels caudal along the left side.

**Figure 2:**
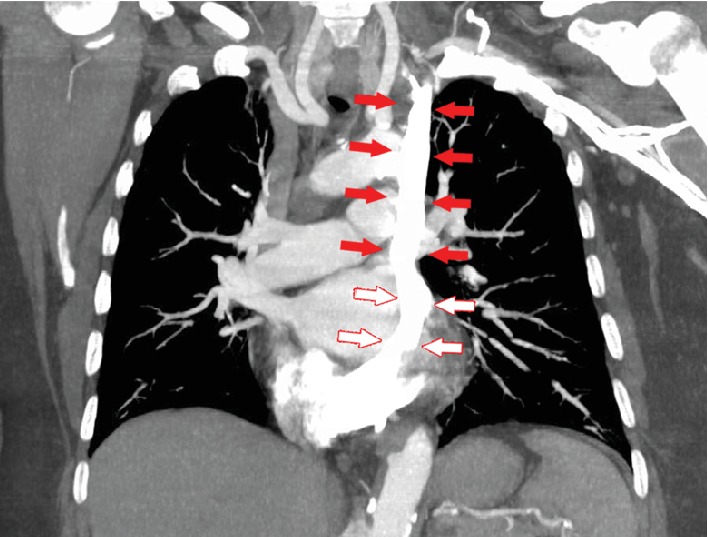
CT angiogram chest that demonstrates duplication of the superior vena cava (red arrows) with a persistent left superior vena cava, which drains into the coronary sinus (red outline arrows) and then the right atrium.

A 62-year-old female with a recent diagnosis of invasive ductal carcinoma on a right breast core needle biopsy presented for elective mediport placement for initiation of chemotherapy. A left-sided mediport placement was attempted. Intraoperatively, the left subclavian vein was accessed, the wire was placed, and fluoroscopy showed the wire to be abnormally travelling caudal on the left side without crossing midline (Figure 1). Because placement of the wire into the SVC could not be verified, the wire was taken out and the subclavian vein was again reaccessed with the same fluoroscopic results. The procedure was aborted, and a CT angiogram of the chest was obtained (Figure 2), revealing that the patient had a duplicate superior vena cava draining into the coronary sinus. The mediport was then placed on the right side via cannulation of the right subclavian vein two days later without complication.

## 3. Discussion

The superior vena cava (SVC) drains blood from the upper half of the body and is where central venous catheters of the upper half of the body are placed. The SVC measures 7 cm in length and ends in the right atrium after making a slight curve posterior and to the right side. Embryologically, the SVC is formed by three different embryonic vein segments, which increases the likelihood to develop SVC anomalies. The right SVC arises from the right precardinal vein and right common cardinal veins, and the left SVC arises from the left precardinal and left common cardinal veins. These veins constitute the initial main venous drainage system of the primitive heart. Normally, an anastomosis will form between the left and right venous systems to create the left brachiocephalic vein. Meanwhile, the left precardinal vein and left common cardinal vein will atrophy to form normal adult vasculature. The failure of the left anterior cardinal vein, proximal to the brachiocephalic anastomosis, to involute results in a duplicate superior vena cava [1–3]. A persistent SVC occurs in 0.3% to 0.5% of the general population and up to 4.5% of patients with other associated cardiac anomalies [1, 2, 4].

In patients with a duplicate superior vena cava, 82% of patients have a normal right-sided SVC with a persistent left SVC. In 90% of cases, the left superior vena cava drains into the coronary sinus followed by the right atrium, and thus, the patient is typically asymptomatic and does not require any treatment. In these cases, placement of central venous lines and pacemakers can cause irritation of the coronary sinus and result in hypotension, arrhythmia, myocardial ischemia, and cardiac arrest. In the remaining 8% to 10% of the cases, a persistent left SVC may drain into the left atrium, forming a right-to-left shunt which can give risk to systemic air or particulate emboli from catheter use and places the patient at an increased risk of developing right-sided heart failure [1, 2, 4, 5]. Therefore, it is imperative to know the drainage of the duplicate superior vena cava prior to use.

Suspicion of a persistent superior vena cava occurs when the catheter or wire takes an abnormal left paramedial intrathoracic course during a chest X-ray or, as seen in this case, under fluoroscopy [1, 2, 4]. Definitive imaging is determined by cross-sectional CT or MR angiogram of the chest. Ultrasound and transthoracic echocardiogram can identify catheters in the right atrium, but are not standard of practice [1]. If there is suspicion for persistent left superior vena cava catheterization, it is safer to determine the drainage via cross-sectional CT or MR imaging than immediately removing the catheter and reattempting recannulation. The differential diagnoses for a left paramediastinal catheter on chest X-ray include introduction of the catheter in the left internal mammary, left superior intercostal, or left pericardiophrenic vein, as well as the catheter placement intrapleurally, intrapericardially, or possibly within a great vessel. The consequence of removing a catheter within the aforementioned areas is potential uncontrolled hemorrhage [2, 6–10].

## 4. Conclusion

Knowledge of the most common venocaval anatomical variations, such as a duplicate superior vena cava, and how to appropriately manage these patients in the presence of a central venous catheter placement is vital to prevent and appropriately manage potential complications.
